# Effect of Physical-Sports Leisure Activities on Young People’s Psychological Wellbeing

**DOI:** 10.3389/fpsyg.2020.543951

**Published:** 2020-10-21

**Authors:** Ana Eva Rodríguez-Bravo, Ángel De-Juanas, Francisco Javier García-Castilla

**Affiliations:** National University of Distance Education, Madrid, Spain

**Keywords:** young people, wellbeing, autonomy, sports, leisure

## Abstract

This article examines the impact of physical-sports activities on the psychological wellbeing of Spanish and Colombian young people. Scientific literature highlights that young people devote leisure time to sports for the purpose of enjoyment, and to maintain good health and body image. In addition, it provides the opportunity to socialize, and come into contact and connect with people who have shared interests. It is also an ideal resource for learning and developing social skills to ensure inclusion and the appropriate strategies for emotional management. Similarly, it favors the learning of values that promote the assumption of responsibilities, decision-making capacity, tolerance to frustration, and the development of resilience. This study considers the inherent benefits of physical-sports activities in order to analyze the impact on young people’s assessment of their own psychological wellbeing. To this effect, a quantitative ex post facto study was designed, and Ryff’s Model of Psychological Wellbeing was used with 1,148 young people from Spain and Colombia aged 16–21. The young people were asked whether or not they performed any type of physical-sports activity in their leisure time and the type of activity performed. The results show that young people who perform such activities have higher overall levels of psychological wellbeing. In turn, they emphasize that the perform of physical-sports activities has a positive impact on three of the dimensions of psychological wellbeing: *self-acceptance, positive relations with others*, and *purpose in life*. In addition, significant differences in young people’s psychological wellbeing were found depending on whether they perform individual, team or other physical-sports activities. These results provide a basis for the proposal and design of interventions with young people based on sports and leisure activities as socio-educational strategies.

## Introduction

The concept of wellbeing is strongly rooted in the field of *humanistic psychology* and in the more recent perspective of *positive psychology*. Key studies in the field of wellbeing have been performed by Allport (1958, 1961), Rogers (1961), Erikson (1963, 1982), Deci and Ryan (1985), Ryff (1989, 1991), Ryff and Keyes (1995), Ryff and Singer (1996, 2008), Seligman (1999), Seligman and Csikszentmihalyi (2000) and Ryan and Deci (2001), inter alia. The concept has been studied from two different perspectives, hedonic and eudaimonic (Deci and Ryan, 1985, 2008; Ryff and Singer, 2008; Waterman et al., 2010; Adler and Seligman, 2016). The principal components of the hedonic perspective link wellbeing with happiness, affection and life satisfaction. Whereas the eudaimonic perspective relates happiness to the development of human potential via activities that enable the individual to become involved and fulfilled (Romero et al., 2009). Subjective wellbeing and psychological wellbeing are outlined as the most representative constructs of both perspectives (Keyes et al., 2002). Recent studies show the existence of differences between both perspectives of wellbeing through the manifestation of positive individual traits (gratitude, love, hope, curiosity, enthusiasm, etc.) in relation to subjective wellbeing and psychological wellbeing (Hausler et al., 2017).

This article looks at psychological wellbeing from a eudaimonic perspective described by Ryff (1995) as one’s effort to perfect and develop one’s own potential. Consequently, wellbeing is understood as a multidimensional construct that involves personal and social aspects that influence the way we understand, interpret, and are in the world, as well as how we face life’s events and challenges. This implies that psychological wellbeing relies on the employment of emotions and strategies whose appropriateness and effectiveness depend on an individual’s optimal physical and social functioning (Romero et al., 2009). Understood in this way, wellbeing refers to the enhancement of one’s own living conditions through the development and improvement of personal capacities and skills to face life’s challenges and stages (Cardona et al., 2014).

### Physical Sports Activities and Psychological Wellbeing

Over the last two decades, the relationship between physical-sports activities and psychological wellbeing has attracted the attention of the scientific community. Numerous studies have demonstrated that a direct relationship exists between both variables (Babyak et al., 2000; Sale et al., 2000; Cantón, 2001; Chen, 2001; Romero et al., 2009; Reigal et al., 2014; González-Hernández et al., 2017). In addition, all types of physical-sports activities have been identified as favoring positive psychological wellbeing (Penedo and Dahn, 2005) regardless of the environment in which they are preformed (Lawton et al., 2017).

More specifically, regularly performed physical activity is associated with high levels of life satisfaction, quality of life and happiness (Stubbe et al., 2007) and the development of structures and resources for the enjoyment of a stable and balanced life (Candel et al., 2008; Thompson et al., 2011). In turn, performing physical activity through frequent and regular sports programs or independent activities, is related to improvements not only in wellbeing but also in mental health, autonomy, memory, body image, optimism, emotional clarity, and mental flexibility (Garrido et al., 2011).

However, the benefits of physical activity on psychological wellbeing are dependent on the attitudes and behaviors that individuals have toward physical activity and sports (Cuadra-Martínez et al., 2012). Positive attitudes and behaviors toward performing physical activity conditions people’s perceptions of their own health and psychological wellbeing (Mackay et al., 2011). In this regard, positive relationships based on indicators of persistence, psychological wellbeing and self-efficacy give rise to a combination that encourages personal growth and increases satisfaction via the effort involved when performing physical activity (González-Hernández et al., 2017).

### Physical Sports Activities and Psychological Wellbeing in Young People

Taking as a reference the theoretical model on the construct of wellbeing proposed by Ryff (1989, 1991) based on six theoretical dimensions (*self-acceptance; positive relations with others; autonomy; environmental mastery; personal growth*, and *purpose in life*), it has been observed that young people tend to excel in two dimensions, *positive relations with others* and *personal growth* (Mayordomo et al., 2016; Meléndez et al., 2018). In contrast, they tend to score lower than other age groups in *self-acceptance, autonomy*, and *environmental mastery* (Meléndez et al., 2018), variables that are more characteristic of maturity (Allport, 1961), which tends to evolve positively with age (Ryff, 1989, 1991; Ryff and Keyes, 1995).

By the same token, it has also been shown that there is a direct correlation between young people’s levels of psychological wellbeing and their coping strategies. Young people with high levels of psychological wellbeing use coping strategies aimed at problem solving and managing related emotions. In contrast, young people with low levels of wellbeing tend to use passive and avoidant forms of coping (Romero et al., 2009). In turn, young people’s psychological wellbeing also seems to be related to their dominant values. In this regard, Bojanowska and Piotrowski (2019) identified four types of relationships: (1) young people with predominate values of openness and personal improvement, and high levels of *autonomy* on the psychological wellbeing scale; (2) young people with values of openness and self-transcendence, who score high in *autonomy* and *positive relations with others*, (3) young people with more traditional values linked to preservation and self-transcendence, whose wellbeing is linked to *personal growth* and *positive relations with others*; and (4) young people who lack clear dominant values, who present lower levels of *autonomy* and *personal growth*. Psychological wellbeing in young people seems to be positively associated with the existence of a stable set of goals, values and norms (Berzonsky and Cieciuch, 2016).

Furthermore, physical activity is the most popular of all the activities young people dedicate their leisure time to (De-Juanas et al., 2018; Fragüela et al., 2018; García-Castilla et al., 2018). Moreover, it is a practice that young people link to perceptions of self-determination and high levels of satisfaction (Codina et al., 2018). They regard physical-sports activity as a positive aspect in their lives, owing to its potential to promote values linked to their psychological wellbeing, along with relaxation, responsibility, commitment and personal satisfaction (Ponce de León Elizondo et al., 2009). Likewise, participation in sports activities, especially in team or individual competitive sports, is very popular among young people. Various studies have reported the benefits of these activities during adolescence for the development of self-concept, improved social skills and self-esteem (Bowker, 2006; Zimmermann-Sloutskis et al., 2010; Eime et al., 2013; Gotova, 2015). Similarly, sport leads to the acquisition of life skills and healthy habits recommended by the World Health Organization (2010).

However, there are numerous studies that confirm that active participation in physical-sports activities also improves psychological wellbeing, especially in those areas closely related to the management of emotions (Baker and Brownell, 2000; Biddle, 2000; Kerr and Kuk, 2001; Leith, 2002; Hellison, 2003). On the whole, it has been observed that physical activity and sport are related to wellbeing, as shown in the study by Greenleaf et al. (2014) performed with more than 1,400 young students in the United States, and the more recent study by McMahon et al. (2017) performed in ten European countries with more than 11,000 adolescents. In the latter, positive correlations were found between the frequency of physical activity and wellbeing in both adolescent males and females. In their study of 493 vulnerable young people, García-Castilla et al. (2016) found that practicing sports leads to benefits such as the promotion of values including teamwork, cooperation, interpersonal relations, and greater development of autonomy, which, in turn, enables young people to acquire better life skills (Días et al., 2000; Marques et al., 2013). In a study of 589 young people with an average age of 24 who regularly practice sports, González-Hernández and Valadez (2016) found positive correlations between personality, coping, and motivation. More recently, Bou et al. (2020), with a sample of 266 young people with an average age of 13, found that regular sports practice has a direct impact on self-esteem.

## Materials and Methods

### Specific Objectives

A quantitative, descriptive, cross-sectional ex post facto study was performed, the main objective being to observe whether there were differences in young people’s psychological wellbeing according to whether or not they showed a preference for performing physical-sports activities. This first objective draws on the hypothesis that the psychological wellbeing of young people who perform physical-sports activities, as part of their valuable leisure time, is different to those who do not perform any type of physical-sports activity. The other important aspect of this study is its second objective: to characterize young people’s psychological wellbeing according to the types of physical-sports activities they prefer to perform. Moreover, this study addresses the hypothesis that the psychological wellbeing of the young people who perform physical-sports activities differs according to the type of activity they prefer to perform. In relation to this, this paper also presents partial results on the physical-sports activities of young people from a comprehensive study on psychological wellbeing and autonomy in the transition to adulthood.

### Participants

The general sample used in the study consisted of 1,148 young people with a minimum age of 16 and a maximum age of 21, and an average close to the age of majority (*M* = 18.20; SD = 1.80). The study sample were primarily recruited from secondary schools and universities. Young people in employment also participated. As an exclusion criterion, it was decided not to include those individuals who had functional, physical or mental difficulties that prevented them from participating in the study. The study was performed from late 2018 to early 2019. The study was conducted using an intentional non-probability sample of young people from Bogotá (55.7%; Colombia) and Madrid (44.3%, Spain). Of the total, 60.3% were female and 39.7% were male. Regarding the results, 59.7% of the young people studied stated they regularly performed physical-sports activities while 40.3% stated that they did not perform any physical-sports activities whatsoever. In parallel, we worked with a subsample comprised of the group of young people who stated that their preferential leisure activity was physical sports (*n* = 676), which corresponds to 67.5% of the general sample. The answers provided by this group to the question “What is your favorite type of physical-sports activity?” gave rise to the following categories: 34.8% (*n* = 235) showed a preference for “Team sports,” 30.6% (*n* = 207) “Individual sports,” and 34.7% (*n* = 225) “Other physical-sports activities.”

### Models

In this study, the Ryff Psychological Wellbeing Scale adapted to the Spanish population by Díaz et al. (2006) was used. This adaptation is based on Ryff’s (1989) multidimensional model and evaluates psychological wellbeing. Consequently, it looks at wellbeing from a eudaimonic perspective (Vera-Villaroel et al., 2013). This model is widely used by international researchers (Ryan and Deci, 2001; Lindfors et al., 2006; Abbott et al., 2010; De-Juanas et al., 2013), and responds to a Likert-type rating scale with six response alternatives, where 1 is totally disagree and 6 is totally agree. The scale comprises 39 items based on a six-dimensional model: the capacity to evaluate oneself and one’s own past while maintaining a positive attitude toward the self despite one’s limitations (*self-acceptance*, α = 0.83); the capacity to establish and maintain quality relationships with others based on trust (*positive relations with others*, α = 0.81); the capacity to maintain one’s individuality with self-determination in the face of diverse adversities, contexts and situations (*autonomy*, α = 0.73); the ability to efficiently direct and control one’s life to generate a favorable environment and satisfy needs and desires (*environmental mastery*, α = 0.71); the ability to evolve and continuously develop as a person, while continuing to grow through positive learning (*personal growth*, α = 0.68), and lastly, the factor that measures an individual’s psychological functioning is the ability to set goals that are aligned with one’s beliefs about the purpose, meaning and significance of life (*purpose in life*, α = 0.83).

Furthermore, in this study a brief ad-hoc questionnaire was used to collect sociodemographic data on the participants (age, gender, place of origin, etc.), who were also asked about their preferred leisure activity.

### Procedure and Data Analysis

The models were distributed to the participants in a printed format to be completed by hand in a single session, during school hours in education centers and during rest periods in work centers. The survey took approximately 45 min. Participation was voluntary. The individuals who agreed to participate read the form before giving their informed consent. No incentives were offered to the respondents. Participants and their legal guardians were informed of the purpose of the study and were provided with a set of guidelines. Following approval by the human research ethics committees from the participating universities, the Declaration of Helsinki (64th WMA, Brazil, October 2013) was followed to ensure confidentiality of responses and avoid selection bias.

Once the field work was complete, the database was digitized, and descriptive analyses were designed and performed to statistically represent the sample. In order to examine the differences between the young people who stated that their preferred leisure pursuit was physical-sports activities and those who did not, two different MANOVAs were then performed after ensuring that the assumptions of normality and homoscedasticity were met. A new categorical variable (group) was created from the recoding of the responses to the survey in order to characterize and analyze the differences in the psychological wellbeing of the participants who stated that performed various types of physical-sports activities. For the first MANOVA, the preference for performing physical sports was considered as the independent variable and two groups G1 (people that perform physical activity) and G2 (participants that do not perform physical activity) were constructed and compared. For the second MANOVA, the preferred type of activity was considered as an independent variable and three groups of participants were constructed and associated with the three different types of physical-sports activities that participants could choose as preferred: G1 (sports activities involving cooperation and opposition by different teams were categorized as “Team sports”); G2 (individual and combative sports activities categorized as “Individual sports”); and G3 (artistic and expressive physical activities, activities in the natural environment and other unspecified physical sports activities, categorized as “Other physical-sports activities”).

To evaluate the relationship between dependent variables, correlation analyses between the different dimensions of the Ryff Psychological Wellbeing Scale were performed prior to enter de main analysis. All the statistical analyses were performed using SPSS 25.0 for Macintosh (IBM^®^ SPSS^®^ Statistics 25). Confidence level was set at 95% (*p* < 0.05).

## Results

Table 1 shows descriptive statistics (mean scores and standard deviations) for each of the factors on the psychological wellbeing scale for each group and the total sample.

**TABLE 1:** Descriptive statistics and Student’s *t*-test on psychological wellbeing based on preference for performing physical-sports activities or not.

	Preference for performing physical-sports activities							
	Yes		No					
	M	SD	M	SD	d.f.	t	ρ	*d*
Self-acceptance	25.70	5.59	24.34	5.85	1130	3.927	0.000	0.238
Positive relations with others	25.82	6.14	25.00	6.12	1130	2.211	0.027	0.134
Autonomy	34.16	6.87	34.19	6.78	1130	−0.084	0.933	0.003
Environmental mastery	25.53	5.06	25.00	5.23	1130	1.689	0.092	0.102
Personal growth	32.74	5.20	32.69	5.32	1130	0.180	0.857	0.009
Purpose in life	27.51	5.39	26.37	6.15	1130	3.266	0.001	0.200
Total psychological wellbeing	171.47	25.16	167.32	27.34	1130	2.624	0.010	0.158

Prior to enter the MANOVAs, a correlation analysis between dependent variables was performed to determine the relationship pattern between the dimensions of the psychological wellbeing scale. The correlation matrix for each pair of dimensions of the psychological wellbeing scale, both for the total sample and for each of the sport-preference groups, yielded a very similar pattern. All correlation coefficients were positive and statistically significant (*p* < 0.001) and did not differ between groups. Table 2 shows the resulting correlation matrix between each pair of psychological wellbeing dimensions for the total sample (for the sake of simplicity and clarity, correlations between dimensions within the different groups are not shown as they follow the same pattern as the total sample).

**TABLE 2:** Descriptive statistics and results of the variance analysis for the three groups of physical-sports activities on the dimensions of psychological wellbeing.

	Total		Team sports (G1) *n* = 235		Individual sports (G2) *n* = 207		Other physical-sports activities (G3) *n* = 225					Multiple comparisons
								
	M	SD	M	SD	M	SD	M	SD	*F*	ρ	*f*	
Self-acceptance	25.75	5.57	25.14	5.04	26.51	6.18	25.68	5.45	3.380	−0.035	0.100	G2 > G1 (*ρ* = 0.048)
Positive relations with others	25.89	6.11	24.49	6.10	26.99	5.79	26.33	6.17	10.312	0.000	0.174	G2 > G1 (*p* = 0.000)
												G3 > G1 ρ = 0.004)
Autonomy	34.17	6.89	33.60	7.00	35.22	6.43	33.81	7.12	3.515	0.030	0.102	G2 > G1 (ρ = 0.050)
Environmental mastery	25.55	5.06	24.55	4.92	26.13	4.90	26.08	5.21	7.274	0.001	0.146	G2 > G1 (ρ = 0.004)
												G3 > G1 (ρ = 0.004)
Personal growth	32.73	5.22	31.62	5.52	33.61	4.89	33.09	5.02	9.024	0.000	0.162	G2 > G1 (ρ = 0.001)
												G3 > G1 (ρ = 0.000)
Purpose in life	27.5	5.39	26.74	5.52	28.32	5.18	27.55	5.35	4.785	0.009	0.120	G2 > G1 (ρ = 0.000) G3 > G1 (ρ = 0.000)
Total psychological wellbeing	171.62	25.14	166.16	24.35	176.79	24.49	172.56	25.51	10.360	0.000	174	G2 > G1 (ρ = 0.000)
												G3 > G1 (ρ = 0.000)

In order to compare young people’s psychological wellbeing scores according to their preferences for performing physical-sports activities, a MANOVA was performed with group (G1, G2) as the independent variable, to determine the statistical differences between groups in each dimension of the wellbeing scale. The results of the MANOVA, showed statistically significant differences in psychological wellbeing between both groups of physical-sports activity [*V* = 0.021, *F*(6,1121) = 4.03, *p* = 0.001, η*2p* = 0.02]. The follow-up univariate tests yielded statistically significant differences in three dimensions of the wellbeing scale: *self-acceptance* [*F*(1,1121) = 13.267; *p* < 0.001, η*2p* = 0.012], *positive relations with others* [*F*(1,1121) = 3.77; *p* = 0.052, η*2p* = 0.003], and *purpose in life* [*F*(1,1121) = 4.03; *p* < 0.001, η*2p* = 0.02].

Second, a new MANOVA was performed to compare the scores on psychological wellbeing between the different types of physical-sports leisure activities that young people prefer to perform (G1, G2, G3). The results indicated the existence of statistically significant differences in psychological wellbeing between the three groups, [*V* = 0.062, *F*(12,1316) = 3.538, *p* < 0.001, η*2p* = 0.031]. The univariate analyses showed that significant differences appeared along the 6 dimensions of the psychological wellbeing scale: *self-acceptance* [*F*(2,662) = 3.183; *p* = 0.042, η*2p* = 0.010], *positive relations with others* [*F*(2,662) = 10.311; *p* < 0.001, η*2p* = 0.03], *autonomy* [*F*(2,662) = 3.651; *p* = 0.026, η*2p* = 0.011], *environmental mastery* [*F*(2,662) = 7.078; *p* = 0.001, η*2p* = 0.021], *personal growth* [*F*(2,662) = 8.769; *p* < 0.001, η*2p* = 0.026], and *purpose in life* [*F*(2,662) = 4.678; *p* = 0.010, η*2p* = 0.014].

Last, to follow up the univariate analysis after finding significant differences between groups in all the dimensions of the well-being scale, *post hoc* comparisons (Bonferroni and Games-Howell correction for equal and unequal homogeneity of variance cases) were performed in order to determine between which groups the differences arise in each dimension. Thus, in *self-acceptance* there were significant differences between the “Individual sports” and the “Team sports” groups (*p* = 0.038). Those who practice individual sports showed a greater level of self-acceptance relative to those that practice team sports. In the *positive relations with others* dimension, there were significant differences between the “Individual sports” group and both the “Team sports” group (*p* < 0.001), and the other physical-sports activities group (*p* = 0.004). The highest scores were obtained by those who practice individual sports followed by the “Other physical-sports activities” group and the “Team sports” group. In the *autonomy* dimension, again a statistically significant difference was observed between the “Individual sports” and the “Team sports” groups (*p* = 0.038), indicating that those who practice individual sports showed greater autonomy.

In the *environmental mastery* dimension, the “Team sports” group obtained scores that were significantly below the “Individual sports” and the “Other physical-sports activities” groups (both ps = 0.004). Similar results were obtained for *personal growth*, with the “Team sports” group showing the lower mean, compared to both “Individual sports” (*p* = 0.000) and “Other physical-sports activities” (*p* = 0.008) groups.

Finally, in the *purpose in life* dimension significant differences were found only between the “Individual sports” and the “Team sports” groups (*p* = 0.070), with the higher scores in the former group.

## Discussion

In order for leisure activities to be considered positive and as having constructive potential, they must have the following characteristics: be a healthy activity, integrate positive experiences, and promote social capital and competencies for life (Navarro et al., 2018). In this regard, physical activity is one of the most valued sources of positive and constructive leisure activities, given the physical, psychological and social benefits it provides (World Health Organization, 2010; Garrido et al., 2011; Cuadra-Martínez et al., 2012; Ponce de León Elizondo et al., 2015).

The findings from this study confirm that young people with a preference for performing physical-sports activities in their leisure time present higher average scores in all the dimensions of wellbeing compared to those who do not perform any type of physical-sports activity. These data are congruent with those provided by other studies that have found a direct relationship between both variables (Babyak et al., 2000; Sale et al., 2000; Cantón, 2001; Chen, 2001; Penedo and Dahn, 2005; Romero et al., 2009; Greenleaf et al., 2014; Reigal et al., 2014; González-Hernández et al., 2017; McMahon et al., 2017).

Similarly, the findings in this study are also aligned with studies that identify *positive relations with others* as one of the main strengths of young people’s psychological wellbeing (Mayordomo et al., 2016; Meléndez et al., 2018). They also emphasize the potential of physical-sports activities as a strategy to promote the early development of other dimensions such as *autonomy* and *self-acceptance*, which are directly linked to having a more mature personality (Allport, 1961), which has been documented as improving with age (Ryff, 1989, 1991; Ryff and Keyes, 1995).

This is also related to the degree of self-confidence one has when practicing sport, whether individual or team activities (Martínez-Romero et al., 2016). The findings confirm and qualify the results obtained by Penedo and Dahn (2005) by corroborating that young people’s preference for performing all types of physical-sports activities influences their positive psychological wellbeing, while identifying that the choice of “Individual sports” has a greater influence than “Team sports” and “Other physical-sports activities.” Likewise, the preference for “Other physical-sports activities” also seems to have more influence than “Team sports.” Notwithstanding, there are differences in psychological wellbeing depending on whether an individual practices an individual or team sport. González-Hernández and Valadez (2016) found the lowest indicators of affability and motivational orientation (ego) in individual sports, and the lowest indicators of open-mindedness and motivational orientation (task) in team sports. In turn, Almeida (2017), in a sample of 95 subjects with an average age of 25, found significant differences between subjective levels of post-exercise recovery, revealing higher levels of stress in those subjects practicing individual sports. However, Méndez (2017) found that in a sample of 329 participants that those who practice individual sports have higher levels of emotional intelligence than those who compete in team sports.

In short, it can be concluded that physical activity is a key source of valued leisure-time among young people, which has a high potential to promote their positive psychological wellbeing and, consequently, contribute to their self-realization and personal satisfaction (Valdemoros San Emeterio et al., 2016). Given that young people attach great importance to leisure activities in their daily lives (Rodríguez-Bravo et al., 2018), encouraging the performance of physical activity can contribute to stimulating dimensions of psychological wellbeing in which they excel (*positive relations with others* and *personal growth*) and reinforce others in which they fall behind (*self-acceptance, autonomy* and *environmental mastery*).

To this end, we believe it is essential to develop programs that use physical-sports activities in intervention strategies with young people, given the opportunities they provide to socialize, and come into contact and connect with people who have shared interests (De-Juanas et al., 2018; García-Castilla et al., 2018). Physical-sports activities are an ideal resource for developing self-concept and self-esteem, learning and developing social skills that favor social inclusion, appropriate emotional management strategies, values that promote the assumption of responsibilities, decision-making capacity, tolerance to frustration and the development of resilience (Hartmann, 2003; Bowker, 2006; Zimmermann-Sloutskis et al., 2010; Eime et al., 2013; Buelens et al., 2015; Chen et al., 2015; Gotova, 2015; Fernández-Gavira et al., 2018).

In order for such programs to improve young people’s wellbeing they must be based on social relationships that promote autonomy, participation, and also structure activities. Moreover, the educators/trainers responsible for their development must be trained in such aspects (Haudenhuyse et al., 2012, 2013; González et al., 2015; McDavid et al., 2017). In turn, such programs must offer young people opportunities to develop competencies linked to emotional intelligence (Di Fabio and Kenny, 2016) and to assess and reflect on their values and long-term goals (Berzonsky and Cieciuch, 2016). It should be highlighted that intervention programs must be supported and sustained by adequate funding, in order for the performance of physical-sports activities to really undertake the function of a mechanism for social inclusion and not just become another means of social exclusion (Collins and Haudenhuyse, 2015).

## Limitations and Future Research

The data reported in this paper may constitute a unique reference framework for the sample studied in Madrid and Colombia. However, although the sample size is considerable, it should be noted that conducting a purposing sampling among a group of young people in Spain and Colombia may offer a specific insight into the results that limit their generalization to other populations. Having said that, we believe it is important to replicate the study with other European and Latin American populations. We also believe it is important to explore the psychological wellbeing of young people and their preference for physical-sports activities throughout their lives and not only in a cross-sectional study. In addition, we are aware that the objectives we proposed in this study did not contemplate the use of other measures that could be used to perform other analyses (for example: values, personality, etc.). We believe that this may be of great interest to future research.

## Data Availability Statement

All datasets presented in this study are included in the article/supplementary material.

## Ethics Statement

The studies involving human participants were reviewed and approved by Ethics Committee of the Universidad Santo Tomás (Bogotá, Colombia) and the Ethics Committee of the Universidad Nacional de Educación a Distancia (Madrid, Spain) and is, therefore, in accordance with the Declaration of Helsinki (seventh revision 2013, Fortaleza, Brazil). The participants provided their written informed consent to participate in this study.

## Author Contributions

AR-B coordinated the project. ÁD-J set up the database and completed the statistical analysis. AR-B and FG-C drafted the initial version of the article, which was then revised by all three authors. AR-B and FG-C prepared the introduction and theoretical framework and wrote the discussion section and AR-B also reviewed the references section. All authors contributed to the article and approved the submitted version.

## Conflict of Interest

The authors declare that the research was conducted in the absence of any commercial or financial relationships that could be construed as a potential conflict of interest.
